# Health‐related quality of life of esophageal cancer patients in daily life after treatment: A multicenter cross‐sectional study in China

**DOI:** 10.1002/cam4.1817

**Published:** 2018-10-22

**Authors:** Qian Liu, Hongmei Zeng, Ruyi Xia, Gang Chen, Shuzheng Liu, Zhiyi Zhang, Yuqin Liu, Guizhou Guo, Guohui Song, Yigong Zhu, Xianghong Wu, Bingbing Song, Xianzhen Liao, Yanfang Chen, Wenqiang Wei, Wanqing Chen, Guihua Zhuang

**Affiliations:** ^1^ Department of Epidemiology and Biostatistics, School of Public Health Xi'an Jiaotong University Health Science Center Xi'an China; ^2^ Xi'an Center for Disease Control and Prevention Xi'an China; ^3^ National Cancer Center/Cancer Hospital, Chinese Academy of Medical Sciences and Peking Union Medical College Beijing China; ^4^ Flinders Health Economics Group, School of Medicine Flinders University Adelaide South Australia Australia; ^5^ Henan Cancer Hospital Zhengzhou China; ^6^ Wuwei Cancer Hospital of Gansu Province Wuwei China; ^7^ Gansu Provincial Cancer Hospital Lanzhou China; ^8^ Linzhou Cancer Hospital Linzhou China; ^9^ Cixian Institute for Cancer Prevention and Control Cixian Cancer Hospital Handan China; ^10^ Luoshan Center for Disease Control and Prevention Xinyang China; ^11^ Center for Disease Control and Prevention of Sheyang County Yancheng China; ^12^ Tumor Prevention and Treatment Institute Harbin Medical University Harbin China; ^13^ Hunan Provincial Cancer Hospital Changsha China; ^14^ Yueyang Lou District Center for Disease Prevention and Control Yueyang China

**Keywords:** China, EQ‐5D, esophageal cancer, health‐related quality of life, utility

## Abstract

**Background:**

The improvement of diagnostic and therapeutic techniques has prolonged the survival time of patients with esophageal cancer. Little is known, however, about their health‐related quality of life (HRQoL) in daily life after treatment.

**Methods:**

Esophageal cancer patients who had been discharged from hospitals more than one year and healthy controls identified by screening were recruited from seven study centers covering eastern, central, and western regions of China. Patients were categorized into severe dysplasia/carcinoma in situ and stages I, II, III, and IV cancer, respectively. The EQ‐5D was employed to assess HRQoL. Multivariate regression analyses were conducted.

**Results:**

A total of 1456 patients and 2179 controls were recruited. After adjusting for potential confounding factors, the likelihood of reporting problems in the five dimensions of patients was 3.8 to 23.1 times higher than controls, whilst the mean EQ‐5D utility score was 0.311 (95% CI, 0.276‐0.346) lower than controls. The mean utility scores of each patient subgroup were 0.158, 0.289, 0.303, 0.296, and 0.505 (95% CIs: 0.108‐0.208, 0.243‐0.336, 0.261‐0.346, 0.244‐0.347, and 0.437‐0.573) lower than controls, respectively. Patients had the greatest impairment in the self‐care dimension compared with controls, followed by the usual activities dimension. Therapeutic regimen, duration of illness, other chronic disease status, age, and marital status also had significant impact on different aspects of HRQoL in patients.

**Conclusions:**

Esophageal cancer significantly impaired patients' HRQoL in daily life after treatment. Advanced cancer stages were associated with larger decrements on health state utility. Utility scores reported here can facilitate further cost‐utility analyses.

## 1 INTRODUCTION

Esophageal cancer is the world's eighth most common cancer and the sixth leading cause of cancer‐related deaths with around 80% of the cases occurring in developing countries.^1^, ^2^ In China, the incidence and mortality of esophageal cancer rank fifth and fourth of all cancers, respectively.^3^ The improvement of diagnostic and therapeutic techniques has prolonged the survival time of patients with esophageal cancer. It is crucial to understand esophageal cancer patients’ physical and psychological health in their daily life after treatment, which will benefit health management services.

As a multidimensional construct describing individuals’ perceptions of their physical, psychological, and social functioning, health‐related quality of life (HRQoL) can holistically assess health outcomes than clinical parameters, particularly important in chronic diseases. Existed studies in China reported that esophageal cancer patients’ HRQoL significantly reduced one month after treatment and recovered to pretreatment level 12 months after treatment^4^; personal characteristics were associated with the patients’ HRQoL.^5^ These studies enrolled a small patient sample in a particular region, and so the generalizability of conclusions is limited. More studies from abroad focused on the impact of surgery or other treatments on the HRQoL of esophageal cancer patients.^6^, ^7^, ^8^ There have also been some studies used the time trade‐off (TTO) method to assess the health state utility of esophageal cancer patients according to the disease progression.^9^, ^10^, ^11^

Our study further contributes to the literature by evaluating the HRQoL of esophageal cancer patients in their daily life after treatment. In particular, it aims to report health state utility scores associated with different cancer stages that can further facilitate the calculation of quality‐adjusted life‐years for economic evaluations. Currently, very limited empirical evidence is available on the health state utility of esophageal cancer internationally. In this study, a large‐scale multicenter population survey was conducted using the EQ‐5D questionnaire, one of the most widely used generic preference‐based instruments internationally to measure HRQoL. Another advantage of choosing EQ‐5D is that by using the recently published Chinese‐specific tariff,^12^ the health state utility scores which reflect the preferences of Chinese population can be derived.

## 2 MATERIALS AND METHODS

### 2.1 Subjects

In 2015, we launched a project aiming to evaluate the efficacy and feasibility of endoscopic screening for upper gastrointestinal cancer in both high‐risk and non‐high‐risk areas of China. Totally, 140 000 participants were enrolled in a cluster randomized trial. The information of overall project design and study locations was shown in detail previously.^13^ Briefly, the seven study centers are located across eastern, central, and western regions of China. Of them, three are in areas with high risk of upper gastrointestinal cancer (including Wuwei City, Linzhou County, and Cixian County), and the other four are in non‐high‐risk areas (including Harbin City, Changsha City, Sheyang County, and Luoshan County). In high‐risk areas, all the subjects in intervention group were screened by endoscopy. In non‐high‐risk areas, about 30% of the subjects in intervention group identified through a questionnaire were screened by endoscopy.

The current study was part of this project conducted in the same areas using a case‐control design. In each study center, we aimed to collect 150 patients with esophageal cancer and 50 or 20 patients with esophageal severe dysplasia/carcinoma in situ (CIS), considering the fact that less patients with severe dysplasia/CIS were identified in non‐high‐risk areas. The eligible cases were those who received inpatient treatment for the main diagnosis of esophageal cancer or esophageal severe dysplasia/CIS and had been discharged from the seven screening centers more than one year by the time of the survey. They were identified from the hospital management information system in each study center and were divided into five subgroups according to the American Joint Committee on Cancer Staging System (7th ed.): severe dysplasia/CIS, stage I, stage II, stage III, and stage IV cancer. The above sample size can completely meet what is required for each subgroup (n = 150) calculated according to the literature.^14^ To facilitate the calculation of sample size, the mean health state utility score of esophageal cancer patients published from previous literature was used (mean = 0.84, standard deviation = 0.22).^15^ It is further expected that the results are within ±0.05 of the true mean health state utility score. For the healthy controls, we aimed to recruit 300 individuals from each study center in the upper gastrointestinal cancer screening cohort. We used a stratified random sampling framework (stratified by age and gender) in each study center to select the healthy controls, that is the screening negative individuals. The survey was conducted between 1 October 2016 and 31 March 2017.

The study was approved by the independent ethics committee of National Good Clinical Practice Center for Anticancer Drugs, National Cancer Center, Chinese Academy of Medical Sciences and Peking Union Medical College (2015SQ00223). The study protocol was registered in Chinese Clinical Trial Registry (ChiCTR‐EOR‐16008577). All subjects signed the informed consent before participating this study.

### 2.2 Questionnaire

Basic information included socio‐demographic and clinical characteristics (eg, clinical stage, duration of illness, therapeutic regimen) was obtained through accessing electronic medical records or the information registered when screening.

HRQoL was assessed using the Chinese version three‐level EQ‐5D questionnaire. This brief instrument contains six questions: the first five questions constitute a descriptive system of five dimensions (mobility, self‐care, usual activities, pain/discomfort, and anxiety/depression); the 6th question is a visual analogue scale (VAS) (on which “100” corresponds to “best imaginable health status” and “0” corresponds to “worst imaginable health status”). The five dimensions and corresponding three response levels (“no problems,” “moderate problems,” and “extreme problems”) generate 243 possible health states. Telephone interview, an acceptable alternative to face‐to‐face interview, was adopted to collect EQ‐5D questionnaire, and respondents were asked to respond to the questionnaire based on their own perception on the day of the survey. The EQ‐5D was scored using a validated Chinese population‐specific value set developed using the TTO technique.^12^ The theoretical utility scores ranged from −0.149 to 1, of which 1 represents full health, 0 represents death, and negative values represent states worse than death.

### 2.3 Statistical analysis

Descriptive analyses were firstly conducted. Comparisons between subgroups were analyzed by using appropriate test statistics according to the variable and its distribution. Multivariate analysis based on the percentage of reporting problems (either moderate or extreme problems) of each dimension was analyzed by binary logistic regression. Tobit regression was used for multivariate analysis based on the EQ‐5D utility score owing to the ceiling effect of the EQ‐5D.^16^ Multivariate linear regression was used to analyze characteristics that were associated with the EQ‐VAS score. *P* < 0.05 (two‐sided) was statistically significant. All data were analyzed by STATA 11.0 (STATA Corp., College Station, TX, USA).

## 3 RESULTS

### 3.1 Respondents’ characteristics

A total of 5153 esophageal cancer patients and 3698 healthy controls were tried to contact by telephone in the seven study centers. Of esophageal cancer patients, 1652 could not get in touch because they went somewhere else or changed their telephone numbers, 1952 had died and 93 refused to participate. Within controls, 1318 could not be contacted and 201 refused the interview. The final study sample consists of 2179 people in the control group, and 1456 esophageal cancer patients, including 257 cases of severe dysplasia/CIS, 313 cases of stage I, 381 cases of stage II, 288 cases of stage III, 204 cases of stage IV cancer, and 13 patients with a clinical stage classified as “unknown.”

Table 1 presents descriptive statistics of respondents by disease status. Patients and controls had mean ages of 64.27 and 54.74 years, respectively. More than 90% of patients had a duration of illness of 1 to 4 years, and more than 70% of patients had no other chronic diseases. Significant differences were observed between patients and controls in all characteristics but marital status.

**Table 1:** Socio‐demographic and clinical characteristics of respondents

	Esophageal cancer patients						Controls (n = 2179)
	Severe dysplasia/CIS (n = 257)	Stage I (n = 313)	Stage II (n = 381)	Stage III (n = 288)	Stage IV (n = 204)	Total[Link] (n = 1456)	
Age (y, %)[Link]							
≤55	14.0	14.7	12.9	16.0	13.7	14.1	52.0
56‐60	15.6	14.4	15.0	12.5	11.3	13.9	17.8
61‐65	33.9	30.0	24.1	26.0	27.9	28.1	17.0
≥66	36.5	40.9	48.0	45.5	47.1	43.9	13.2
Gender (%)[Link]							
Male	67.7	73.5	76.4	76.7	74.0	73.8	49.8
Female	32.3	26.5	23.6	23.3	26.0	26.2	50.2
Marital status (%)							
Married	96.1	94.9	95.8	96.2	94.1	95.5	94.5
Others	3.9	5.1	4.2	3.8	5.9	4.5	5.5
Occupation (%)[Link]							
Farmer	88.3	82.4	79.0	77.4	77.5	81.0	N/A
Nonfarmer	11.7	17.6	21.0	22.6	22.5	19.0	
Duration of illness (y, %)							
1	46.7	41.2	36.2	43.4	36.3	40.7	N/A
2‐4	46.3	52.1	57.0	52.8	56.8	53.1	
≥5	7.0	6.7	6.8	3.8	6.9	6.2	
Other chronic diseases (%)							
No	77.0	77.6	75.1	77.8	75.5	76.6	N/A
Yes	23.0	22.4	24.9	22.2	24.5	23.4	
Therapeutic regimen (%)[Link]							
Surgery	38.5	29.1	21.8	20.1	13.7	24.7	N/A
Radical resection	46.3	30.6	19.4	24.3	2.0	24.8	
Radiotherapy	2.7	11.8	12.6	15.3	27.9	14.1	
Chemotherapy	1.6	5.1	15.5	12.2	24.5	11.3	
Surgery plus adjuvant chemotherapy	0.4	3.5	6.0	4.5	3.4	3.8	
Concurrent chemoradiotherapy	0.4	6.4	10.0	13.9	9.8	8.2	
Symptomatic treatment	4.3	10.9	13.4	6.6	16.2	10.2	
Others	5.8	2.6	1.3	3.1	2.5	2.9	
Study center (%)^b,c^							
Cixian County	25.7	23.9	16.8	13.9	14.7	18.8	15.8
Wuwei City	24.5	23.0	24.4	11.1	5.4	18.6	13.9
Linzhou County	21.4	9.9	10.5	14.2	18.1	14.9	14.2
Harbin City	7.8	9.3	14.7	21.9	16.2	13.8	14.0
Changsha City	3.9	13.1	13.6	16.3	14.7	12.4	14.5
Sheyang County	7.8	11.2	12.3	12.8	15.2	11.7	13.8
Luoshan County	8.9	9.6	7.7	9.8	15.7	9.8	13.8

N/A, controls did not collect or did not have these characteristics.

Including 13 patients with a clinical stage classified as “unknown.”

Statistical difference between esophageal cancer patients and controls (*P* < 0.05).

Statistical difference among subgroups of esophageal cancer patients (*P* < 0.05).

### 3.2 Respondents’ HRQoL

Table 2 shows respondents’ HRQoL measured using the three‐level EQ‐5D. It can be seen that the proportion of respondents who had problems in any one of the five dimensions was significantly higher in esophageal cancer patients than controls (all *P* < 0.001). The mean EQ‐5D utility and EQ‐VAS scores of patients were significantly lower than controls (0.81 vs 0.96 and 72.22 vs 85.14, respectively, all *P* < 0.001). For both patients and controls, respondents had the highest odds of reporting problems in the pain/discomfort dimension, followed by the anxiety/depression dimension. Among patients, the likelihood of reporting problems in the mobility, self‐care, and usual activities dimensions significantly increased along with the disease state progression (all *P* < 0.05), whilst the mean EQ‐5D utility and EQ‐VAS scores significantly decreased (from 0.90 to 0.66 and from 80.56 to 62.17, respectively, all *P* < 0.05).

**Table 2:** EQ‐5D outcomes of esophageal cancer patients vs healthy controls

	Percentage of reporting problems in each dimension					Utility score (mean ± SD)[Link] ^,^ [Link]	EQ‐VAS score (mean ± SD)[Link] ^,^ [Link]
	Mobility[Link] ^,^ [Link]	Self‐care[Link] ^,^ [Link]	Usual activities[Link] ^,^ [Link]	Pain/discomfort[Link]	Anxiety/depression[Link]		
Controls	2.8	1.1	2.0	15.8	10.1	0.96 ± 0.09	85.14 ± 10.78
Esophageal cancer patients	25.3	21.5	29.8	50.3	34.4	0.81 ± 0.24	72.22 ± 18.41
Severe dysplasia/CIS	10.9	8.6	17.6	34.6	19.1	0.90 ± 0.16	80.56 ± 13.15
Stage I	23.6	20.4	24.6	50.8	34.2	0.82 ± 0.23	73.70 ± 16.17
Stage II	22.6	19.2	32.3	55.4	38.6	0.81 ± 0.21	72.17 ± 16.77
Stage III	29.2	22.9	32.4	48.3	32.9	0.80 ± 0.24	69.93 ± 18.65
Stage IV	46.1	41.2	45.6	63.2	48.8	0.66 ± 0.34	62.17 ± 23.97

The utility and EQ‐VAS scores between esophageal cancer patients and controls were compared using Mann‐Whitney *U* test.

Statistical difference between esophageal cancer patients and controls (*P* < 0.001).

Statistical correlation between clinical stage and outcome variables in esophageal cancer patients (*P* < 0.05).

**Figure 1:**
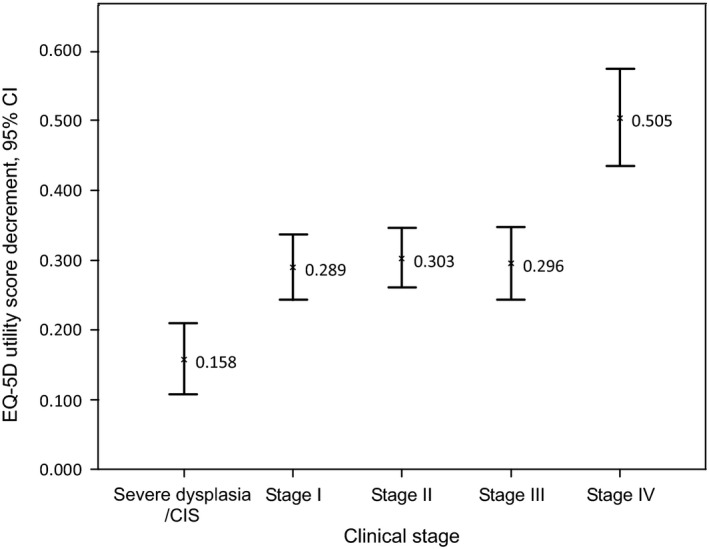
Decrements on the EQ‐5D utility score of esophageal cancer patients vs controls

Controlling for potential confounding factors (ie, age, gender, marital status, and study centers), multivariate regression results showed that the percentages of reporting problems in the five dimensions of patients were 10.6, 23.1, 17.9, 5.1, and 3.8 times higher than those of controls (all *P* < 0.001), whilst the mean EQ‐5D utility and EQ‐VAS scores were 0.311 (95% CI, 0.276‐0.346) and 10.859 (95% CI, 9.766‐11.953) lower (all *P* < 0.001). These results suggested that patients had the greatest impairment in the self‐care dimension compared with controls, followed by the usual activities dimension. The key regression result on the EQ‐5D utility scores is shown in Figure 1, with the key variable of interest (clinical stage) on the horizontal axis and the absolute magnitude of decrement on utility score on the vertical axis. It can be seen that compared to controls, the mean utility scores of patients were significantly lower, with decrements of 0.158, 0.289, 0.303, 0.296, and 0.505 (95% CIs: 0.108‐0.208, 0.243‐0.336, 0.261‐0.346, 0.244‐0.347, and 0.437‐0.573) for severe dysplasia/CIS, stage I, stage II, stage III, and stage IV cancer, respectively (all *P* < 0.001). In addition, among these clinical stages, the magnitudes of decrement on the EQ‐5D utility score were not significantly different among stages I to III.

### 3.3 Factors associated with HRQoL among esophageal cancer patients

Binary logistic regression analyses on each of the five dimensions show that after controlling for potential confounding factors, there were significant associations between cancer clinical stage and the odds of reporting problems in all the five dimensions—patients in more advanced clinical stages were more likely to report having issues on HRQoL dimensions (Table 3). The other two clinical characteristics were also significant explanatory variables: duration of illness in the mobility dimension and therapeutic regimen in all the five dimensions. Among socio‐demographic characteristics included in the regression, age was significant in the pain/discomfort dimension (with patients aged 61‐65 years significantly more likely to report having issues than the reference group who were aged 55 years or younger), whilst marital status was significant in the mobility, self‐care, and usual activities dimensions (with married patients significantly less likely to report having issues in the above three dimensions). A set of study center dummies were also significant in regressions.

**Table 3:** Binary logistic regression on the percentage of reporting problems in the five dimensions of EQ‐5D among esophageal cancer patients

Variable	Mobility OR (95% CI)	Self‐care OR (95% CI)	Usual activities OR (95% CI)	Pain/discomfort OR (95% CI)	Anxiety/depression OR (95% CI)
Clinical stage, reference group: severe dysplasia/CIS					
Stage I	2.360^**^ (1.390, 4.007)	2.699^**^ (1.528, 4.770)	1.166 (0.730, 1.864)	1.516^*^ (1.035, 2.221)	2.220^**^ (1.445, 3.411)
Stage II	1.908^*^ (1.104, 3.299)	2.263^**^ (1.253, 4.089)	1.629^*^ (1.015, 2.613)	1.690^**^ (1.146, 2.492)	2.561^**^ (1.659, 3.954)
Stage III	4.005^**^ (2.277, 7.044)	4.015^**^ (2.175, 7.410)	2.266^**^ (1.375, 3.732)	1.532^*^ (1.010, 2.325)	2.508^**^ (1.574, 3.996)
Stage IV	8.269^**^ (4.454, 15.350)	10.341^**^ (5.299, 20.179)	3.741^**^ (2.157, 6.488)	2.983^**^ (1.844, 4.826)	5.269^**^ (3.145, 8.828)
Duration of illness (y), reference group: 1 y					
2‐4	1.202 (0.892, 1.619)	1.052 (0.769, 1.438)	0.980 (0.740, 1.297)	0.965 (0.752, 1.237)	1.067 (0.824, 1.382)
≥5	1.991^*^ (1.108, 3.576)	1.309 (0.704, 2.434)	1.393 (0.808, 2.401)	1.034 (0.620, 1.723)	1.656 (0.996, 2.753)
Therapeutic regimen, reference group: surgery					
Radical resection	0.713 (0.418, 1.218)	0.974 (0.555, 1.709)	0.892 (0.547, 1.453)	1.436 (0.969, 2.127)	1.141 (0.743, 1.752)
Radiotherapy	1.225 (0.703, 2.135)	1.261 (0.704, 2.257)	1.879^*^ (1.120, 3.150)	2.146^**^ (1.366, 3.372)	1.250 (0.779, 2.004)
Chemotherapy	0.682 (0.380, 1.223)	0.757 (0.404, 1.422)	1.011 (0.587, 1.743)	1.848^*^ (1.160, 2.944)	0.992 (0.605, 1.628)
Surgery plus adjuvant chemotherapy	1.000 (0.442, 2.263)	0.941 (0.369, 2.398)	2.118^*^ (1.024, 4.381)	3.822^**^ (1.849, 7.901)	1.535 (0.755, 3.118)
Concurrent chemoradiotherapy	0.692 (0.373, 1.285)	0.992 (0.524, 1.879)	1.484 (0.837, 2.632)	1.990^**^ (1.186, 3.340)	1.733^*^ (1.023, 2.935)
Symptomatic treatment	1.457 (0.820, 2.588)	1.401 (0.766, 2.562)	2.007^*^ (1.162, 3.467)	3.116^**^ (1.845, 5.263)	1.367 (0.816, 2.292)
Others	0.048^**^ (0.006, 0.370)	0.073^*^ (0.009, 0.570)	0.124^**^ (0.028, 0.551)	0.181^**^ (0.067, 0.489)	0.132^**^ (0.030, 0.575)
Age (y), reference group: ≤55 y					
56‐60	1.048 (0.611, 1.798)	1.172 (0.646, 2.126)	1.054 (0.625, 1.778)	1.289 (0.837, 1.986)	0.953 (0.600, 1.514)
61‐65	0.993 (0.622, 1.584)	1.211 (0.725, 2.024)	1.492 (0.958, 2.325)	1.666^**^ (1.140, 2.437)	1.047 (0.703, 1.559)
≥66	1.238 (0.791, 1.939)	1.600 (0.978, 2.618)	1.426 (0.927, 2.194)	1.147 (0.795, 1.653)	1.195 (0.814, 1.755)
Marital status, reference group: married					
Others	3.412^**^ (1.770, 6.577)	2.994^**^ (1.510, 5.936)	2.970^**^ (1.596, 5.527)	1.570 (0.881, 2.797)	1.235 (0.684, 2.229)

In addition to what have been reported in the table, a set of study center dummies have also been included. Other respondents’ characteristics which were included in the regression but statistically insignificant (*P* > 0.05) include gender, occupation, and other chronic disease status.

*P* < 0.05;

*P* < 0.01.

The regression results reported in Table 4 show that controlling for potential confounding factors, patients in more advanced cancer stages were significantly associated with lower utility and EQ‐VAS scores. Compared to those who had severe dysplasia/CIS, the magnitudes of mean EQ‐5D utility decrement ranged from −0.109 (stage I cancer) to −0.328 (stage IV cancer), whilst the mean EQ‐VAS decrement ranged from −3.784 (stage I cancer) to −13.087 (stage IV cancer). In addition, therapeutic regimen also had significant impact on the EQ‐5D utility/EQ‐VAS score. Other significant variables include marital status (with the utility scores of married patients significantly higher), other chronic disease status (only significant in the EQ‐VAS score), and a set of dummy variables representing study centers.

**Table 4:** Multivariate analysis on the EQ‐5D utility score and the EQ‐VAS score of esophageal cancer patients

Variable	EQ‐5D utility score *β* (95% CI)	EQ‐VAS score *β* (95% CI)
Clinical stage, reference group: severe dysplasia/CIS		
Stage I	−0.109^**^ (−0.166, −0.051)	−3.784^**^ (−6.464, −1.104)
Stage II	−0.114^**^ (−0.172, −0.055)	−5.025^**^ (−7.741, −2.309)
Stage III	−0.143^**^ (−0.208, −0.080)	−7.453^**^ (−10.374, −4.533)
Stage IV	−0.328^**^ (−0.407, −0.249)	−13.087^**^ (−16.440, −9.734)
Other chronic diseases, reference group: no		
Yes	−0.033 (−0.079, 0.013)	−2.918^**^ (−5.013, −0.823)
Therapeutic regimen, reference group: surgery		
Radical resection	−0.043 (−0.106, 0.019)	−3.013^*^ (−5.885, −0.141)
Radiotherapy	−0.123^**^ (−0.197, −0.049)	−7.653^**^ (−10.990, −4.315)
Chemotherapy	−0.066 (−0.138, 0.006)	−3.433^*^ (−6.856, −0.010)
Surgery plus adjuvant chemotherapy	−0.145^**^ (−0.246, −0.044)	−3.923 (−8.958, 1.112)
Concurrent chemoradiotherapy	−0.111^*^ (−0.197, −0.026)	−5.484^**^ (−9.266, −1.702)
Symptomatic treatment	−0.133^**^ (−0.208, −0.058)	−6.042^**^ (−9.719, −2.366)
Others	0.403^*^ (0.232, 0.574)	8.693^**^ (3.395, 13.990)
Marital status, reference group: married		
Others	−0.139^**^ (−0.236, −0.042)	−7.857^**^ (−12.069, −3.645)

In addition to what have been reported in the table, a set of study center dummies have also been included. Other respondents’ characteristics which were included in the regression but statistically insignificant (*P* > 0.05) include age, gender, occupation, and duration of illness.

*P* < 0.05;

*P* < 0.01.

## 4 DISCUSSION

In this multicenter study, we investigated the HRQoL of esophageal cancer patients with different stages, using a total of 1456 patients and 2179 healthy controls. To the best of our knowledge, it is the largest sample size in the literature reported so far. We expected that some generalizable conclusions can be drawn, especially for the patients in their context of daily life.

In both esophageal cancer patients and healthy controls, the pain/discomfort was the mostly impaired dimension, followed by the anxiety/depression. This observation is consistent with other studies using the EQ‐5D.^15^, ^17^ Indeed, clinical evidence suggests that pain was one of the major symptoms seriously affecting the HRQoL of cancer patients: depending on the stages of cancer, 25%‐75% of the patients suffered from varying degrees of pain.^18^ Cancer patients also suffered from anxiety and depression issues, and might have higher suicidal tendencies.^19^ The comparisons between patients and controls in our study further suggested that esophageal cancer patients had the greatest impairment in the self‐care dimension, followed by the usual activities dimension.

The mean EQ‐5D utility score of each subgroup of esophageal cancer patients was 0.158, 0.289, 0.303, 0.296, and 0.505 lower than controls, respectively. As expected, larger magnitudes on utility decrement were found in patients with more advanced cancer stages. It is also worth noting that among the four cancer stages, only the mean utility score of the most advanced stage was significantly different from the other three stages. It is difficult to compare the mean utility decrements from this study to others since it is the first time the Chinese tariff has been used in esophageal cancer patients. Gerson et al^9^ found through the TTO exercise that the mean TTO utility decreased from 0.91 (nondysplastic Barrett's esophagus) to 0.85 (low‐grade dysplasia)/0.77 (high‐grade dysplasia) and was the lowest for the scenario of esophageal cancer (0.67, representing a decrement utility of 0.24 from nondysplastic Barrett's esophagus). In another TTO study conducted by McNamee et al,^10^ the mean TTO utility scores for grade 1 (mild) to grade 5 (severe) in esophageal cancer patients decreased gradually (ranging from 0.66 to 0.08). Wildi et al also reported that the utility scores were negatively associated with the severity of esophageal cancer (the mean utility score for nondetectable cancer to metastatic disease ranged from 0.99 to 0.52).^11^ Overall, results from this study and literature all suggest that early diagnosis and treatment of esophageal cancer will facilitate the maintenance of patients’ HRQoL.

Other clinical characteristics which were shown to significantly impact on esophageal cancer patients’ HRQoL include disease duration, other chronic disease status, and therapeutic regimen. The longer duration of illness (≥5 years), the more likely that esophageal cancer patients reported having problems in the mobility dimension, but not in the other four dimensions. Lee et al reported that cancer survivors (including lung, liver, colon, stomach, breast, and cervix cancer) who were diagnosed for ≥5 years had higher odds of reporting problems in mobility and self‐care dimensions.^20^ Abusaad et al reported that breast cancer patients who were diagnosed for ≥30 months had significantly lower physical and role functioning, and more financial difficulties, but had no significant in other aspects (eg, emotional functioning),^21^ similar to our study. The HRQoL of esophageal cancer patients treated with various therapeutic regimens was different. The differences between the results of other studies and ours may stem from different severity of illness and recovery time after treatment.^6^, ^7^, ^8^, ^15^

Among socio‐demographic characteristics, age and marital status had significant effect on the HRQoL of esophageal cancer patients. Elder patients were more likely to report problems in the pain/discomfort dimension. Tomaszewski et al reported that oesophagogastric cancer patients aged ≥60 years had higher pain and discomfort scores than patients aged <60 years.^22^ In general, the HRQoL of elder patients is always significantly lower owing to the decline in physical function and the poor endurance capacity. Married patients were less likely to report problems in the mobility, self‐care, and usual activities dimensions, whereas the utility and EQ‐VAS scores were higher. The study conducted by Miller et al suggested that married esophageal cancer patients reported higher HRQoL in legal concerns and friend and family support than single patients.^23^

This multicenter study has two limitations. Firstly, healthy controls were not completely comparable to patients with regard to the socio‐demographic characteristics. However, regression analyses were conducted to control observable characteristics. In addition, since controls were chosen from the same area like patients, it is less likely that unobservable environmental factors could lead to a bias comparison. Secondly, since the Chinese‐specific EQ‐5D‐3L tariff has just been developed, the minimally important difference is still unknown. As such we cannot draw a conclusion on whether the differences in the EQ‐5D utility score are clinically important or not.

In summary, our study indicated that esophageal cancer significantly impaired patients’ HRQoL in daily life after treatment. Along with the increasing severity of cancer states, larger decrements on health state utility were found. Early diagnosis and treatment are essential in the management of esophageal cancer patients. More emotional and social support should be given to older patients, patients without spouse, and patients with more advanced clinical stages. The health state utility scores reported in this study can facilitate further cost‐utility analyses.

## CONFLICT OF INTEREST

The authors made no disclosures.
